# A new ant-butterfly symbiosis in the forest canopy fills an evolutionary gap

**DOI:** 10.1038/s41598-021-00274-x

**Published:** 2021-10-21

**Authors:** Gabriela Pérez-Lachaud, Franklin H. Rocha, Carmen Pozo, Lucas A. Kaminski, Noemy Seraphim, Jean-Paul Lachaud

**Affiliations:** 1 Departamento de Conservación de La Biodiversidad, El Colegio de la Frontera Sur, Avenida Centenario Km 5.5, 77014 Chetumal, Quintana Roo Mexico; 2 Departamento de Zoologia, Instituto de Biociências, Universidade Federal do Rio Grande do Sul, Porto Alegre, RS Brazil; 3 Instituto Federal de Educação, Ciência e Tecnologia de São Paulo, São Paulo, Brazil; 4 Centre de Recherches sur la Cognition Animale (CRCA), Centre de Biologie Intégrative (CBI), Université de Toulouse, CNRS, UPS, Toulouse, France; 5 Present Address: Departamento de Apicultura, Universidad Autónoma de Yucatán, Campus de Ciencias Biológicas y Agropecuarias, Mérida, Yucatán Mexico

**Keywords:** Behavioural ecology, Biodiversity, Ecological networks

## Abstract

Myrmecophilous butterflies can establish complex symbiotic relationships with ants. A caterpillar wandering among the brood of the aggressive ponerine ant *Neoponera villosa* was found inside the core of a nest built in the myrmecophytic bromeliad *Aechmea bracteata*. This is the first caterpillar found living inside a ponerine ant nest. Its DNA barcode was sequenced, and an integrative approach was used to identify it as *Pseudonymphidia agave*, a poorly known member of the subtribe Pachythonina in the riodinid tribe Nymphidiini. The cuticle of the tank-like caterpillar lacks projections or tubercles and is covered dorsally by specialized flat setae that form an armor of small plates. Ant-organs potentially related to caterpillar-ant signaling, such as perforated cupola organs and tentacle nectary organs, are present. These morphological traits, together with evidence of social integration (direct contact with host brood, protective morphology, slow movement, no host aggressiveness), suggest that *P. agave* is a symbiotic, social parasite of *N. villosa*, preying on its host brood. However, several knowledge gaps remain, including oviposition site, dependence on bromeliad association, steps to colony integration, and larval diet through development. Carnivory has been reported in all known members of the subtribe Pachythonina (caterpillars prey on honeydew-producing hemipterans) suggesting a shift to myrmecophagy inside the ant nests as a possible evolutionary transition.

## Introduction

Animals of several classes, especially arthropods, establish long-term associations with ants and benefit from these facultative or obligate associations in several ways. These so-called myrmecophiles (for a detailed definition, see^1,2^) circumvent the behavioral and chemical defenses of ants and thrive in their homeostatic, stable nests or in their surrounding territories^3,4^. Living in concealed environments –the ant nests–, most specialized myrmecophiles are rarely encountered, restricted to specific habitats or microhabitats. Moreover, they are unevenly distributed in time and space, presenting challenges for their study^5^.

Initially restricted to social insect species that parasitize closely related sister-taxa^3,6,7^, the term ‘social parasite’ has been extended to distantly related organisms^8^. In a very broad sense, all specialized organisms that are equipped to infiltrate insect societies and take advantage of their social host over an extended period of time can be considered social parasites^2,8^. This term has been widely applied to many ant-associated lycaenid and riodinid butterflies^9–11^ but also to other organisms such as microdontine syrphid flies^12–14^ or beetles^2,15–17^. Symbiotic associations between lepidopteran caterpillars and ants are diverse and widespread, spanning at least six lepidopteran superfamilies: Noctuoidea, Papilionoidea, Pyraloidea, Tineoidea, Gelechioidea, and Zygaenoidea^18–20^. These interactions range from commensalism or mutualism to social parasitism, and relationships may be facultative or obligate, occurring during only a portion or the whole life cycle of the myrmecophile^21,22^. These associations are noticeably prominent in two sister butterfly families: Lycaenidae and Riodinidae (Papilionoidea)^18^. Myrmecophilous caterpillars of these families typically form symbioses with ants on host plants where the ants, and sometimes also the caterpillars, harvest liquid food from plants (extrafloral nectar) and exudates from hemipterans (aphids, treehoppers, scale insects, and relatives)^23–25^. In trophobiotic interactions, the caterpillars secrete nutritional food rewards for ants and gain potential protection against predators and parasitoids^23,26,27^.

From this ancestral pattern (phytophagous caterpillars), several Lepidopteran lineages have evolved to exploit the colony resources inside the ant nests, either as social parasites that elicit trophallaxis from workers or as brood predators^28–30^. Although more than 99% of lepidopteran caterpillars are phytophagous, evolutionary transition from feeding on plant parts to aphytophagy has occurred several times, with carnivorous caterpillars found scattered throughout the butterfly phylogeny^11,31^. Predation on ant brood has been documented in Lycaenidae, but not in the Riodinidae; however members of both families exhibit complex myrmecophily with the evolution of specific ant-organs and behavioral strategies to integrate and exploit ant host colonies^11^ such as chemical signaling, vibrational and tactile mimicry, ant-dependent oviposition, release of brood-carrying behavior, trophallaxis and others^32–34^. These obligate social parasite species are completely dependent on the ants to complete their life cycle^35^.

Metalmark butterflies (Riodinidae) are classified into two subfamilies: the exclusively Neotropical Riodininae with *ca.* 1200 described species, and the Nemeobiinae which include both Old-World and Neotropical taxa^36–38^. Trophobiosis with ants in this family has evolved in parallel with the Lycaenidae^11,24,39,40^. According to Espeland et al.^41^, caterpillar-ant association has evolved once in the Lycaenidae and twice in the Riodinidae (in the tribes Eurybiini and Nymphidiini, both Riodininae).

Riodinid immatures for which life histories have been documented are defoliators, nectarivores, lichen feeders, detritivores, exploiters, and predators of hemipterans^39,42^. Approximately 20% of the known Riodinidae species are involved in symbiotic associations with ants^41^. Several recent studies have played a role in unveiling the nature of riodinid-ant associations^11,42–44^, confirming the evolution of social parasitism in this family (cleptoparasitism via trophallaxis with ants inside their nest)^11^. Myrmecophagy, though suspected in several instances, has not been confirmed through field observations^45^. Individuals of several ant subfamilies are frequently observed tending riodinids on host plants. Such associations are recurrent in members of the subfamilies Myrmicinae, Formicinae, Dolichoderinae and Ectatomminae, all of which are territorially dominant ants that harvest animal and plant secretions. On the contrary, associations with riodinids are very uncommon in the subfamily Ponerinae (see^39^ and Supplementary Table S1), and this is also true for associations with lycaenids as only two ponerine species of the genus *Odontomachus* (*O. haematodes* and *O. ruficeps*) have been reported to facultatively attend larvae of *Anthene* (*A. flavomaculatus* and *A. lycaenoides*, respectively) on host plants^46,47^. In a few instances, more intimate and stable relationships between riodinids and their ant hosts have also been described. For example, some riodinid species rest and pupate in shelters constructed by ants, and others have been collected inside the nests of various formicine, myrmicine and dolichoderine species^11,48–51^. However, to the best of our knowledge, there is no record of riodinid or lycaenid larvae living within ponerine ant colonies, which may be related, as has been noted for obligate social parasite species of lycaenids, to the fact that their ant hosts typically share certain attributes such as large colony size, ecological dominance, territorial defense, and monopolization of food resources, traits that are not exhibited by most ponerine ants^52^.

Through extensive fieldwork during which colonies of the Neotropical ponerine ant *Neoponera villosa* were collected in the southern region of the Yucatan Peninsula^53,54^, we recorded a single butterfly caterpillar wandering among the ant brood. In this study we: (1) establish the identity of this symbiont through an integrative approach; (2) present some notes on the natural history and behavior of the caterpillar; (3) describe and illustrate its external morphology; (4) discuss the putative functional significance of morphological traits that may have allowed this species to invade aggressive ant societies; and (5) compare them to other described socially parasitic caterpillars.

## Material and methods

### The ant-plant symbiotic system

*Neoponera villosa* is widely distributed from Mexico to Argentina^55^. This is the largest Mexican ant; workers measure 1.2 to 1.3 cm^56^ and have a powerful and painful sting. They are generalist predators and forage mainly in the canopy collecting liquid carbohydrate food sources^57–59^. Throughout its distribution range, this species nests opportunistically in pre-existing cavities in live or dead trees, hollow branches, or cacao pods^57,60–63^, among other plant cavities. However, in the southern part of the Yucatan Peninsula, Mexico, it preferentially uses the myrmecophytic tank-bromeliad *Aechmea bracteata* as a nest site and very few colonies are established on other plants^53,54,61,64–68^. Founding queens show a clear spontaneous preference for *A. bracteata* material^64^ and workers also significantly prefer this bromeliad over other species during nest relocation^54^.

The tightly intertwined leaves of *A. bracteata* collect rainwater and constitute true water reservoirs that are extremely attractive to a wide range of living organisms, from aquatic life forms to terrestrial invertebrates (especially ants) and even vertebrates^61,65,69–71^. In the *A. bracteata* microcosm, a particularly diverse array of specialized and facultative myrmecophiles, mostly antagonists, establish complex trophic interactions with *N. villosa*^53^. To date, various invertebrates have been recorded in direct association with the brood of this species and can be considered as true myrmecophiles^53^. Three of them are brood parasitoids: an unidentified species of *Kapala* (Hymenoptera: Eucharitidae)^53^, an unidentified species of *Blanchardiscus* (Hymenoptera: Encyrtidae)^68^, and the hoverfly *Hypselosyrphus trigonus* (Diptera: Syrphidae)^67,68^. Two species (the pseudoscorpion, *Chelodamus mexicolens*, and an unidentified tenebrionid beetle possibly in the subfamily Alleculinae) are brood predators, while two mites (an unidentified species of the genus *Oplitis* and an unidentified galumnid species) are phoretic on the host adults or larvae and a third unidentified mite species (of the genus *Cosmolaelaps*) is cleptoparasite on the ant larvae. Finally, two other species (a staphylinid beetle of the genus *Myrmigaster* and a diapriid wasp of the genus *Trichopria*) have unclear relationships but have been found wandering on the cocoons with their antennae in direct contact with the host cocoon surface^53^.

### Sampling

**Figure 1:**
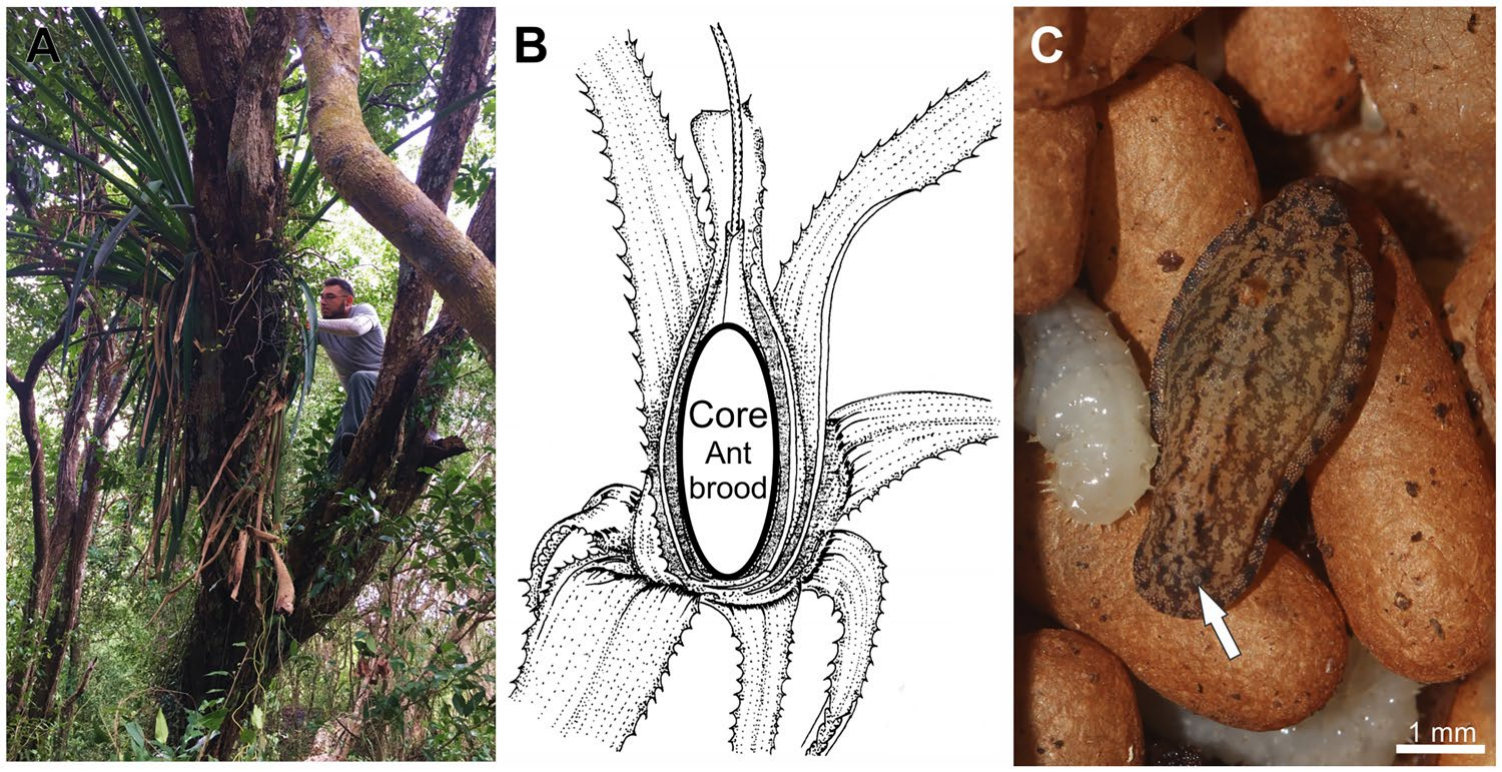
*Pseudonymphidia agave* caterpillar and the ant-plant system. (**A**) Habitat of *Aechmea bracteata* (collector: F.H. Rocha). (**B**) Ant nesting site in the bromeliad (modified from^41^). (**C**) Mature larva of *P. agave* among the ant brood; the arrow indicates the opening of a tentacle nectary organ on the A8 abdominal segment. Photos: (**A**) G. Pérez-Lachaud; (**C**) F. H. Rocha.

Eighty-two colonies of *N. villosa*, all of them nesting in the core of a tank-bromeliad *A. bracteata* (Fig. 1A,B) were collected between January 2016 and April 2018 in several sites in the southern part of the Yucatan Peninsula as part of a larger project^53,54^. *Aechmea bracteata* bromeliads were examined for the presence of *N. villosa* and a ramet of the epiphyte, housing the ants’ colony, was cut off from the supporting branch and dismantled leaf-by-leaf; ants, their brood, and any invertebrate found in the nest were collected and preserved in 96% alcohol.

### DNA barcoding

DNA extraction was performed on two of the thoracic legs of the larva using a standard glass fiber method^72^. Polymerase chain reactions were performed to amplify the mitochondrial cytochrome c oxidase subunit I (COI) gene, using the primers LepF1 and LepR1 developed for Lepidoptera by Hebert et al.^73^, and PCR protocols as in Montes-Ortiz and Elías-Gutiérrez^74^. The PCR products were visualized on a 2% agarose gel (E-Gel 96 Invitrogen), and were sequenced bidirectionally on an ABI 3730XL automated sequencer using LepF1 and LepR1 primers at the Instituto de Biología at the Universidad Nacional Autónoma de México. The sequence was edited using CodonCode v. 3.0.1 (CodonCode Corporation, Dedham, MA, USA) and uploaded to the Barcode of Life Database (BOLD, boldsystems.org) and to GenBank (www.ncbi.nlm.nih.gov; accession number MW627452). We added this larval sequence to a database of published COI barcodes and unpublished sequences (C. Pozo et al., unpubl. data; N. Seraphim, unpubl. data; Supplementary Table S2). Sequences were then combined with a larger matrix of Riodinidae species for a total of 269 specimens comprising all previously sequenced Riodinidae^36,37,41^ including eight nuclear genes as well as the mitochondrial barcode region as described in Seraphim et al.^37^: ArgKin, CAD, GAPDH, Ef1a, IDH, MDH, RpS5, wingless and COI. This expanded matrix was used to obtain a maximum likelihood tree using IQ-TREE software version 1.6.12^75^, with *Curetis barsine* (Lycaenidae: Curetinae) as an outgroup. The model of nucleotide substitution was estimated using the model selection implemented in IQ-TREE^76^. Support was estimated using 10,000 ultrafast boostraps^77^ and the SH-aLRT test^78^. The resulting tree was compressed for clarity.

### Morphology

The caterpillar was examined using a Nikon dissecting stereomicroscope (8–64X) and a JEOL-JSM6010 scanning electron microscope (SEM) equipped with a freezing plate; it was examined under low vacuum at 20 kV and − 31 °C to preserve the integrity of the specimen. Approximate measurements and ratios of measurements were obtained using SEM images. Photos of the larva were taken with a Nikon 850D equipped with a Rodenstok Rodagon 2.8 50 mm lens adapted to a Beseler bellows. A metric scale was included in the photos for calibration. Photos were stacked with HeliconFocus 6.2.2 and processed in Photoshop®. Voucher specimens (the riodinid larva and *N. villosa* ants) were deposited at the Formicidae and Lepidoptera Collections of El Colegio de la Frontera Sur at Chetumal, Quintana Roo, Mexico (ECO-CH-F and ECO-CH-L, respectively). Terminology for the description follows Stehr^79^ for general morphology of the larva and DeVries^80^ for ant-organs.

### Flight period and distribution map

To provide a distribution map and data on adult flight phenology, we compiled distribution data from the literature and from records in the MARIPOSA Data Base at the Museo de Zoología “Alfonso L. Herrera,” Facultad de Ciencias, UNAM, Mexico. This database synthesizes information from Mexican specimens housed in natural history collections around the world. In addition, we used information from the database of the Lepidoptera Collection at ECOSUR and data bases available on internet or published literature for specimens of countries other than Mexico: https://www.gbif.org/ for Honduras, Nicaragua, Trinidad & Tobago; https://www.butterfliesofamerica.com/ for Costa Rica; Godman and Salvin^81^ and D’Abrera^82^ for Panamá; Lamas^83^ for Colombia.

## Results

A myrmecophilous caterpillar (Fig. 1) was found among the ant brood in a *Neoponera villosa* colony collected on June 10, 2017 at Ejido Blasillo, Campeche, Mexico (18°7′13.6056″ N, 89°19′47.791″ W, 263 m asl). The caterpillar was isolated in a small humidified petri dish. After several days, the larva appeared to be in a poor condition and was preserved in alcohol. After this myrmecophilous larva was discovered, intensified collecting efforts uncovered 19 additional *N. villosa* colonies in the same locality^52^, but no additional riodinid larva.

### Caterpillar identification

**Figure 2:**
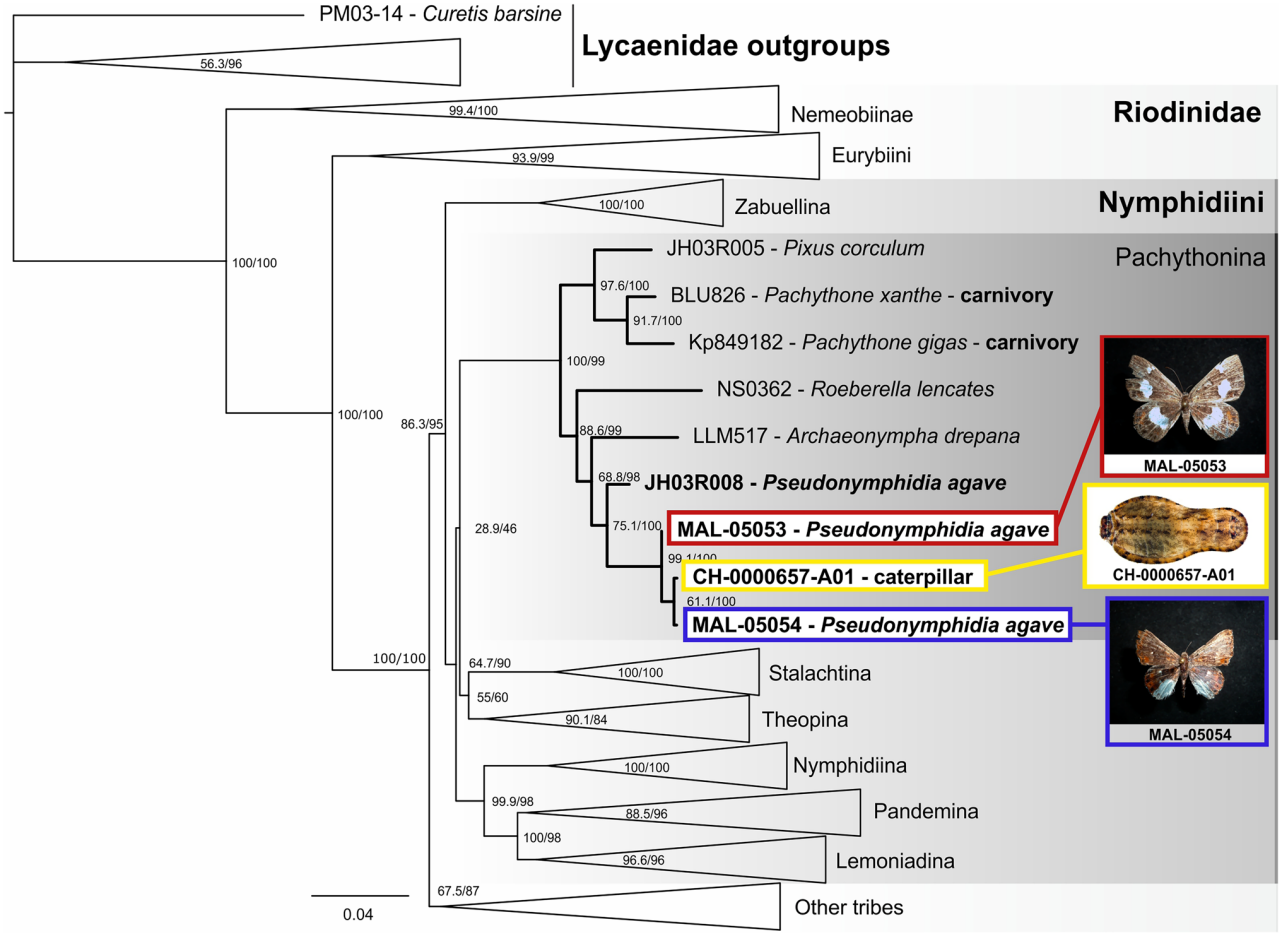
Maximum likelihood tree of the Riodinidae, used to identify the collected larva, showing its systematic position within the subtribe Pachytonina. The remaining clades within the Riodinidae have been compressed for clarity. *Pseudonymphidia agave* sequences are illustrated with photos of the adults: MAL-05053, MAL-05054 (Photos: B. R. Prado-Cuellar) and the caterpillar (Photo: H. Bahena Basave).

We sequenced the COI DNA barcode of the caterpillar and included it with a dataset of other riodinid sequences prior to inferring a phylogenetic tree. This tree placed our caterpillar within the subtribe Pachythonina, sister to *Pseudonymphidia agave* from Mexico (C. Pozo et al., unpubl. data; specimens MAL-05053 and MAL-05054, Fig. 2 and Supplementary Table S2). Indeed, the caterpillar barcode was identical to the barcode of a Mexican *P. agave* adult (MAL-05054), from Calakmul, Campeche, collected in 1998.

*Pseudonymphidia agave* was originally described as *Lemonias agave* Godman and Salvin, 1886. Two *P. agave* subspecies are currently recognized: the nominate subspecies and *leucogonia* (Stichel, 1911). Based on similarities in genital morphology, antennal length and wing pattern, Hall and Harvey^84^ temporarily placed *agave* and *leucogonia* in the genus *Pseudonymphidia*. This genus forms a monophyletic clade along with *Pachythone*, *Roeberella* and *Pixus* in the phylogenetic hypothesis of Seraphim et al.^37^, who erected the subtribe Pachythonina to include several genera placed as *incertae sedis* in previous revisions of the family (see also^36^). In two recent controversial studies, Zhang et al.^85,86^ placed several species of the subtribe Pachythonina in the genus *Pachythone*, including the two currently valid species of *Pseudonymphidia* (*P. agave* and *P. clearista*) (see Figs. 9 and 32 in^85^ and Fig. 30 in^86^), but without considering the distribution nor the diversity or natural histories of the species of the whole genus, and without formally presenting the novel combinations or providing a reason for transferring the two species from the genus *Pseudonymphidia*. Here, we decided not to follow Zhang et al.^85,86^ and use ‘*Pseudonymphidia agave*’ until new concrete integrative taxonomic evidence is presented. According to the phylogenetic tree obtained here (Fig. 2), the closest relative of the genus *Pseudonymphidia* is *Archaeonympha drepana*, a genus not included in the study of Seraphim et al.^37^ that needs to be revised since its type has not yet been sequenced and its other two species are placed in different subtribes within the Nymphidiini (*Archaeonympha urichi* is in the subtribe Theopina according to Zhang et al.^86^).

### Prevalence, natural history, and behavioral observations

Of the 82 *N. villosa* colonies collected between January 2016 and April 2018 around the southern part of the Yucatan Peninsula, only one colony was infested with a riodinid larva, even though 19 of the colonies were from the same locality (Ejido Blasillo) and its surroundings. The caterpillar of *P. agave* was wandering among the host cocoons and larvae in the presence of workers; no aggression was detected during a 3 h observation period. The host colony where the riodinid caterpillar was discovered nested in an *Aechmea bracteata* bromeliad established at a height of 4 m on a *Haematoxylum campechianum* (Fabaceae) tree, situated in a patch of deciduous forest. The colony was composed of 11 dealated queens, three gynes, 165 workers, 265 larvae, 173 pupae (in cocoons), and many eggs. Larvae and pupae were apparently not parasitized by endo- or ectoparasites. Apart from the riodinid larva, very few other myrmecophiles were found in the colony: only the cleptoparasitic mite *Cosmolaelaps* sp. (present on ant larvae, N = 5) and two species of staphylinid beetles (*Myrmigaster* sp. and *Tyropsis* sp., N = 2 and N = 1, respectively) were found in the nest chambers. Additionally, 17 adults of an unidentified species of Nitidulidae (Coleoptera), and 16 pseudoscorpions (*Chelodamus mexicolens*) were found in the nest refuse.

### Morphology of the *Pseudonymphidia agave* caterpillar

**Figure 3:**
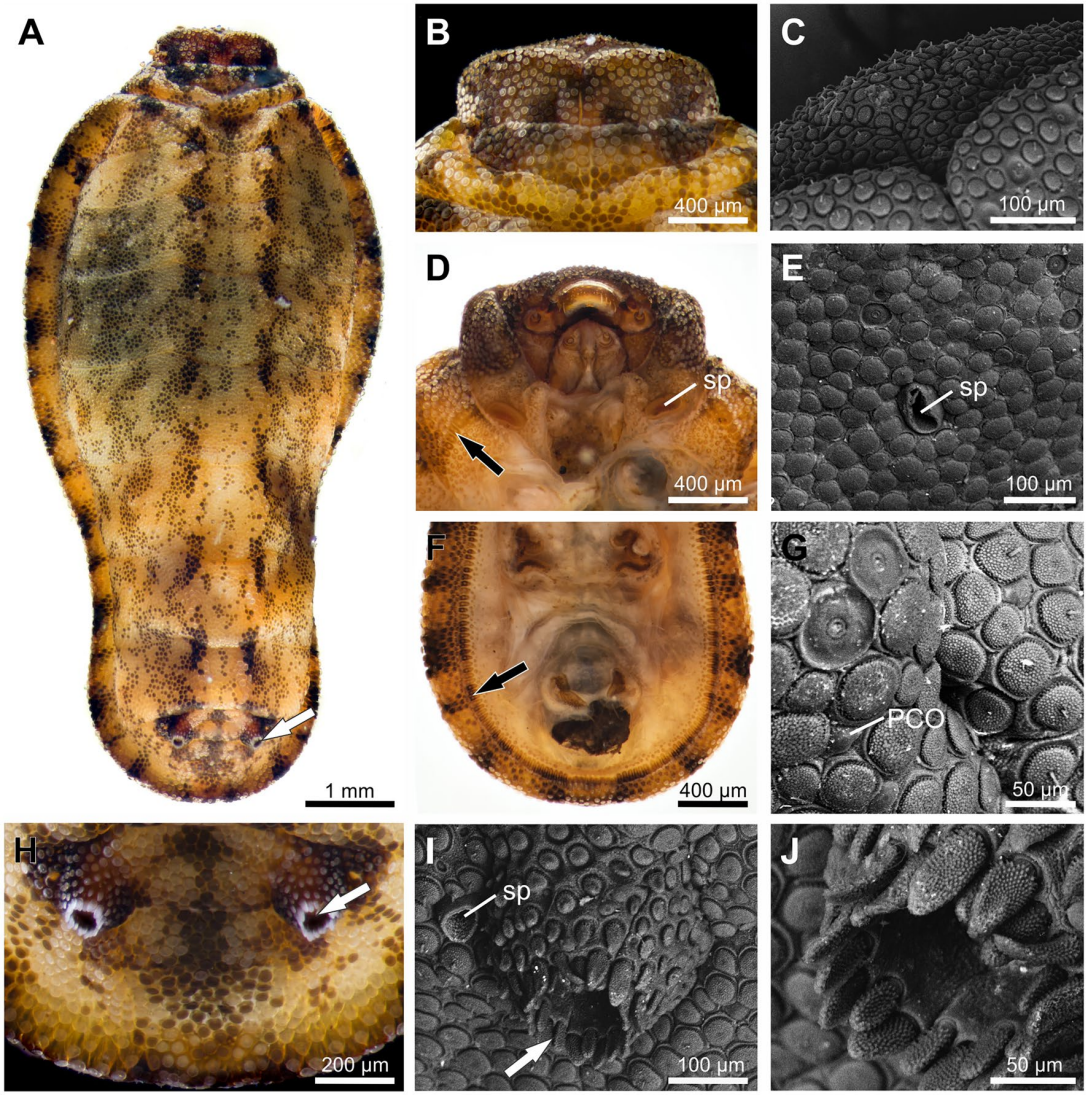
Morphology of the *Pseudonymphidia agave* caterpillar and details in scanning electron microscopy (SEM). (**A**) Dorsal view, the arrow indicates the openings of the tentacle nectary organs (TNOs) on the A8 abdominal segment. (**B**) Bilobed prothoracic shield in dorsal view. (**C**) SEM of the bilobed prothoracic shield. (**D**) Ventral view of the head and thoracic segments, showing the prothoracic spiracle (sp) and fringe of setae (black arrow). (**E**) SEM of the spiracle (sp) on the A3 abdominal segment. (**F**) Ventral view of the last abdominal segments; note lateral fringe of setae (black arrow). (**G**) SEM of the integument covered dorsally by arborescent flat setae and perforated cupola organs (PCO). (**H**) Opening of TNO (arrow); I) and J) SEMs of the opening of TNO (arrow), showing spiracle (sp) and elongated arborescent setae. Photos: (**A**,**B**,**D**,**F**,**H**) H. Bahena Basave; (**C**,**E**,**G**,**I**,**J**) M. Elias-Gutiérrez.

Head capsule width: 2.06 mm, total length: 17.81 mm (N = 1). Head brown; body pale brown with mottled dark spots corresponding to the color of the microscopic setae present in each region (Fig. 3A). Body onisciform without projections or tubercles (small knoblike or rounded protuberances), similar in size to the host cocoons (Figs. 1C,3A). Head and appendages (legs and prolegs) concealed under the body, not visible dorsally (Fig. 3A,D,F). Anterior portion of the body wider than the posterior segments in dorsal view (Fig. 3A). Prothoracic shield bilobed (Fig. 3B). Tegument covered dorsally by arborescent flat setae that form an armor of small plates and perforated cupola organs (PCOs) (Fig. 3C,E,G). Downward pointing setae forming a ventral-lateral fringe (Fig. 3F), and elongated arborescent setae associated with the opening of tentacle nectary organs (TNOs) on A8 (Fig. 3H–J). Prothoracic spiracle located ventrally (Fig. 3D), abdominal spiracles aligned laterally, with exception of those on A2 and A8, which are located subdorsally; openings elevated with elliptical peritrema (Fig. 3I). The larval instar could not be determined with certainty, but from its size it corresponded clearly to an advanced instar.

### Distribution records for *Pseudonymphidia agave* adults

**Figure 4:**
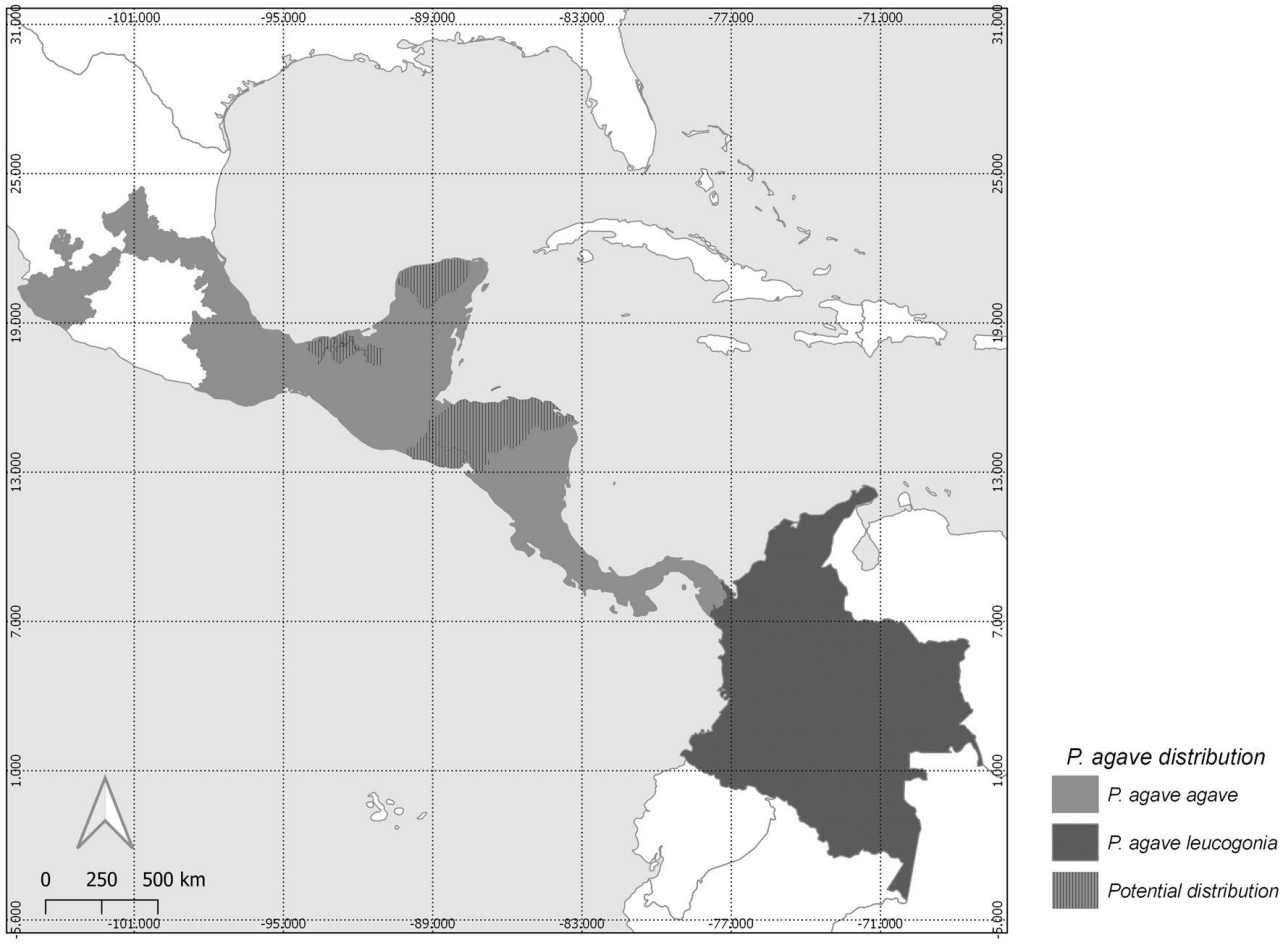
Known distribution of *Pseudonymphidia agave*. Records were compiled from published literature, Museum databases and internet catalogs (see Material and methods for more details). Map created using the free and open source QGIS, version 3.12.0, http://www.qgis.org. Map credit: B. R. Prado-Cuellar.

*Pseudonymphidia agave*, the white-trailed metalmark or agave metalmark, is a rare species, recorded from a few localities. Based on museum specimens, *P. agave* has been recorded from as far north as San Luis Potosi in Mexico and as far south as Colombia (Fig. 4). Specimens have been collected between 0 and 1 600 m^87^. This species seems restricted to specific habitats in tropical forests; adult specimens have been collected in evergreen, semi-evergreen, deciduous, semideciduous, and cloud forests. Adults have been collected on rambutan flowers, *Nephelium lappaceum* (Sapindaceae), in a commercial plantation^87^, but caterpillar host plants are unknown and natural history information was not available prior to this study. Only 52 *P. agave* specimens from Mexico currently exist in museum collections (Supplementary Tables S3, S4). Regarding the museum specimens, flight activities have been recorded almost the whole year, with a significant peak in June and a smaller one in October (Supplementary Fig. S1), a pattern previously observed for the Neotropical riodinids and other small butterflies whereby the greatest number of individuals has been recorded during the wettest season of the year^88^.

## Discussion

The biodiversity of tropical forest canopies is a frontier of knowledge that can still reveal great surprises^89^. In these environments, ants are species rich and abundant, connecting species and trophic levels through antagonistic and mutualistic interactions (reviewed in^90^). Our description of this specific type of biodiversity unveiled an unexpected symbiotic interaction between a butterfly caterpillar and an aggressive ant. Our report that *Pseudonymphidia agave* caterpillars are guests in the nests of *Neoponera villosa* is, to the best of our knowledge, the first case of a butterfly caterpillar in direct contact with the host brood inside a ponerine ant nest. Our finding is the first to provide information on the life cycle of a member of the genus *Pseudonymphidia*, of which the immature stages were previously unknown, and sheds light upon the possible evolutionary steps of social parasitism in riodinids.

In recent years, much new information about the immature stages of Riodinidae has been discovered^11,91–96^. Nevertheless, immatures of some lineages remain completely unknown, principally in the Nymphidiini, suggesting that species are rare or that immature stages have not been found yet because they feed in the canopy. On the other hand, the presence of a *P. agave* larva among the members of a *N. villosa* colony is consistent with the idea that these taxa may exhibit specialized associations with ants, including long periods inside the ant nests^11,39,96^. *Pseudonymphidia agave* belongs to the recently erected subtribe Pachythonina (Nymphidiini), a clade diagnosed on the basis of genetic characters^37^. This group comprises rare and range-restricted taxa associated with tropical forests and is the latest subtribe of Nymphidiini whose natural history information has been published^42^. Species in the Pachythonina for which some life history information is known have armored carnivorous caterpillars specialized in preying on honeydew-producing hemipterans (e.g., *Pachythone* spp.^42,97^) and are thought to carry out ant-mediated oviposition on harmful ant-plant symbioses, using specific ants as oviposition cues (e.g., *Minstrellus grandis*^44^), or preying on ant brood (e.g., *P. agave*; this study). Although scattered, the available information indicates that Pachythonina species demonstrate morphological and behavioral traits allowing coexistence with extremely aggressive ants, including unusual associations with pseudomyrmecine and ponerine ants (Supplementary Table S1). In the miletine lycaenid *Liphyra brassolis* found in the nests of the arboreal formicine *Oecophylla smaragdina*, the tank-like morphology of the caterpillar is considered to be linked to its brutish, myrmecophagous lifestyle^98^. The caterpillar uses its unusual antennae to pull the host brood under the thick cuticle concealing its head and consumes the larvae of its host without being disturbed by the ant workers. However, unlike the unaggressive behavior of *N. villosa* workers confronted to the *P. agave* caterpillar, *L. brassolis* larvae can suffer some aggression by the *O. smaragdina* workers that try to overturn the ant-eating larvae to gain access to their presumably vulnerable underside^98^. Although a commensal or scavenger lifestyle may be considered a preadaptation to stealing food stores or preying on the brood present in ant host nests^19^, the larvae of only a few species in various lepidopteran families (Tineidae, Psychidae, Pyralidae and Noctuidae) are known to eat on food stores or nest material or scavenge on dead organic matter^19,21^. Althought the larvae of some non-myrmecophilous riodinids are detritivores as is the case for *Detritivora barnesi*^39,99^, ant-associated riodinid caterpillars have never been considered as scavengers and the possibility that the larva of *P. agave* that we observed could eat stored food or wastes in *N. villosa* nests seems unlikely.

Ponerine ants are a group of large aggressive ants with a powerful sting^3^. Most species are specialized or generalist predators, occupying a high trophic position^100,101^. The use of liquid foods on foliage, however, has been recorded for some species such as *N. villosa* that harvest liquid secretions from extrafloral nectaries, exudates from honeydew-producing hemipterans and secretions of some facultatively myrmecophilous caterpillars^57–59^ (Supplementary Table S1). According to DeVries^23^ and Eastwood & Fraser^47^ the evolution of symbiosis between caterpillars and ants is associated with ant genera that harvest liquid food on vegetation. The aggressiveness and conspicuous appearance of *N. villosa* ants associated with liquid feeding on plants may have generated an ecological opportunity for the evolution of exploitation by preadapted caterpillars^23,27,102,103^. This hypothesis can be corroborated both in ecological time^104^ and evolutionary time if the specialization between *P. agave* caterpillars on *N. villosa* ants is confirmed.

Detailed behavioral interactions between the *P. agave* caterpillar and *N. villosa* ants, could not be analyzed, and neither tentacle eversion nor liquid or volatile compounds released by the TNOs were observed. However, much evidence suggests that the nature of the relationship between caterpillars of this riodinid and *N. villosa* is not facultative or simply casual: (i) the larva was found among the brood in the core of the ant nest, (ii) it has a general protective morphology, with the head retracted or maintained under the shield-like, thick cuticle of the body much like the ant-brood feeding lycaenid *L. brassolis*^98^, (iii) it had a slow, gradual, slug-like movement behavior, and (iv) the ants did not behave aggressively. These traits indicate that the myrmecophile is well adapted to life inside the ant nest and are consistent with a symbiotic relationship with its host. Exchange of liquid food through regurgitation (stomodeal trophallaxis) is a highly evolved form of social food sharing, but it is infrequent in ponerine ants^58,105^. The first case of social parasitism in riodinids (exploitation of the colony resources through trophallaxis inside the ant host nest) has been recently reported in *Aricoris arenarum* (Riodininae: Lemoniadina)^11^, in nests of the formicine ant *Camponotus punctulatus*, and a similar “cuckoo” life-style could be possible for the *P. agave* caterpillar. However, stomodeal trophallaxis is not known to occur in *N. villosa*^58^; instead, workers share liquid food by means of mandibular pseudo-trophallaxis: workers gather and transport liquid substances with surface tension to maintain a drop between the mandibles. In the nest, they offer the liquid to other workers who “spoon” some of the liquid^58^, but as the head of the *P. agave* larva is protected in a concealed, ventral position, and its rigid armoured cuticle does not allow the larva to bend its body easily, it is unlikely that the caterpillar’s mouthparts could come in contact with the droplet of liquid that is occasionally held by the workers between their mandibles. Since the colony had no other source of food for the caterpillar aside from the ants themselves, and the general rigid morphology of the former likely prevents obtaining food by pseudo-trophallaxis, we further hypothesize that the caterpillar is most probably a brood predator (myrmecophagy), at least during the advanced larval instar as found in our study. Furthermore, in adults of *P. agave*, the wings present a greasy appearance, a trait that has been considered as a potential sign of carnivory^106,107^. Carnivory in riodinid caterpillars has been documented in five Nymphidiini lineages preying on ant-tended hemipterans on plants^42^, but myrmecophagy has only been recorded under artificial conditions^45,108^.

The larva of *P. agave* differs from all other known riodinid caterpillars, though the general tank-like appearance is similar to other riodinids in the Lemoniadina (e.g., *Menander* spp.^39,109^), and Pachythonina subtribes (e.g., *Pachythone xanthe*^42^). Although phytophagous, *Menander* caterpillars are covered with a prominent carapace that flares outward to the substrate and covers the body and legs; they are thus heavily armored and possess a complete set of ant-organs^39,109^. In *P. xanthe* caterpillars, the prothoracic shield divided vertically into two movable plates, the absence of vibratory papillae and a carapace that protects the head and appendages are undoubtedly the most remarkable traits^42^. Some of these larval characters such as the body shape, the morphology of the lateral fringe setae, and the positioning of the spiracles, are shared with *P. agave*, suggesting that they may be morphological synapomorphies for the subtribe. In fact, these morphological characters led us to think that the larva potentially belonged to the subtribe Pachythonina before corroboration through molecular data.

Among myrmecophilous caterpillars, two strategies can be discerned: (i) free-living caterpillars that establish commensal or trophobiotic associations with ants on plants; and (ii) social parasite caterpillars that at some point of their development live within an ant nest^11^. This seems to be related to the progressive activation of ant-organs and the production of putative chemical compounds that trigger ant adoption at a specific moment of the caterpillar development^110^. The life cycle of *P. agave* appears to conform to the latter strategy, but details of the adoption process by the ant colony are unknown. In summary, our data strongly suggest that *P. agave* is an obligate symbiont in *N. villosa* nests and that the advanced larval instar studied here is myrmecophagous. Whether females lay eggs directly on the leaves or inflorescences of the bromeliad is not known but seems possible. As suggested for obligate myrmecophilous lycaenids^111^, the paucity of records of this butterfly might be explained by its highly specialized life history, making the butterfly distribution dependent on the myrmecophytic association between *N. villosa* and the bromeliad *A. bracteata*. Such an assumption seems consistent with the known range of *P. agave*. The range of this species is much narrower than that of its ant host, *N. villosa*, which is consistent with what would be expected of an obligate and host-specific myrmecophilous butterfly, but it extends well beyond the Yucatan Peninsula and, in fact, overlaps perfectly the range of *A. bracteata*, from the 'Huasteca Potosina' area on the Mexican Gulf Coast and the southern part of Sinaloa on the Pacific Coast to northern Colombia^70^.

## Supplementary Information

Supplementary Information.
