# Supplementary material for: A new ant-butterfly symbiosis in the forest canopy fills an evolutionary gap
Source: Sci Rep. 2021 Oct 21;11:20770. doi: 10.1038/s41598-021-00274-x (PMC8531015; doi:10.1038/s41598-021-00274-x)
Supplement: Supplementary file 1 — Supplementary Information. [file 41598_2021_274_MOESM1_ESM.docx]

**Supplementary Information**

**Filling the gap: a new ant-butterfly symbiosis in the forest canopy**

Gabriela Pérez-Lachaud^1,*^, Franklin H. Rocha^1,2^, Carmen Pozo^1^, Lucas A. Kaminski^3^, Noemy Seraphim^4^ & Jean-Paul Lachaud^1,5,*^

^1^ El Colegio de la Frontera Sur, Departamento de Conservación de la Biodiversidad, Avenida Centenario Km 5.5, Chetumal 77014, Quintana Roo, México.

^2^ Present address: Departamento de Apicultura, Campus de Ciencias Biológicas y Agropecuarias, Universidad Autónoma de Yucatán, Mérida, Yucatán, México.

^3^ Departamento de Zoologia, Instituto de Biociências, Universidade Federal do Rio Grande do Sul, Porto Alegre, RS, Brazil.

^4^ Instituto Federal de Educação, Ciência e Tecnologia de São Paulo, Brazil.

^5^ Centre de Recherches sur la Cognition Animale (CRCA), Centre de Biologie Intégrative (CBI), Université de Toulouse, CNRS, UPS, France.

*Authors for correspondence: [igperez@ecosur.mx](mailto:igperez@ecosur.mx); [jean-paul.lachaud@univ-tlse3.fr](mailto:jean-paul.lachaud@univ-tlse3.frx)

Content

Table S1. Published myrmecophilous associations in riodinid butterflies, including tending ants, ant subfamilies, caterpillar feeding habits, notes on type and degree of myrmecophily, localities and references.

Table S2. Sample information of relevant taxa in Fig. 2.

Table S3. Number of specimens in data bases of collections housing *Pseudonymphidia agave* specimens from Mexico.

Table S4. Number of specimens and Mexican states where *Pseudonymphidia agave* individuals have been collected.

Figure S1. Flight activity according to records of Mexican specimens in Museums.

**Table S1.** Published myrmecophilous associations in riodinid butterflies, including tending ants, ant subfamilies, caterpillar feeding habits, notes on type and degree of myrmecophily, localities and references. When possible, records were reassessed based on additional information (L. A. Kaminski, unpubl. data). Records where the ants were not identified were not included. Taxonomic arrangement for Riodinidae follows the phylogenetic hypothesis proposed by Seraphim et al. (2018) and nomenclature of Riodinidae is updated following Seraphim (2019). *: Specialized predacious caterpillars on honeydew-producing hemipterans; **: possible cases of social parasites found inside ant nests.

| **Riodinidae taxon** | **Tending ants** | **Ant subfamilies** | **Caterpillar feeding habits / type and degree of myrmecophily** | **Locality** | **References** |
| --- | --- | --- | --- | --- | --- |
| Eurybiini: Eurybiina | | | | | |
| **Alesa amesis* (Cramer 1777) | *Camponotus femoratus* | Formicinae | Exudates and predation on honeydew-producing hemipterans (HPHs) / trophobiotic, obligate association with parabiotic ants | Peru, Ecuador, Brazil | 1, 2, 3 |
| ***Alesa rothschildi* (Seitz, 1913) | *Cephalotes atratus* | Myrmicinae | Unknown / possibly social parasite, caterpillars with specialized morphology found inside the ant-nest in the canopy | Peru | 4 |
| *Eurybia elvina elvina* Stichel, 1910 | *Neoponera villosa, Camponotus* sp., *Brachymyrmex musculus, Paratrechina* sp., *Pheidole* sp., *Ph.* *gouldi*, *Crematogaster* sp., *Cr.* *sumichrasti*, *Solenopsis* (*diplorhoptrum* group), *S. geminata*, *Wasmannia auropunctata, Ectatomma ruidum, E. tuberculatum* | Ponerinae Formicinae, Myrmicinae, Ectatomminae | Phytophagous on plants / trophobiotic, facultative, including *N. villosa* | Mexico, Costa Rica, Panama | 5, 6, 7, 8 |
| *Eurybia lycisca* Westwood, 1851 | *Neoponera villosa,*  *Camponotus* spp*., Paratrechina* sp., *Crematogaster* sp., *Cr. brevispinosa, Aphaenogaster araneoides*, *Pheidole* sp., *Ph. biconstricta*, *Ph.* nr. *biconstricta*, *Wasmannia auropunctata, Solenopsis* sp., *S.* (*diplorhoptrum* group), *Megalomyrmex foreli, Ectatomma ruidum, E. tuberculatum* | Ponerinae Formicinae, Myrmicinae | Phytophagous on plants / trophobiotic, facultative, including *N. villosa* | Costa Rica, Panama | 6, 7 |
| *Eurybia patrona persona* Staudinger, 1876 | *Neoponera villosa, Camponotus* sp., *Paratrechina* sp., *Crematogaster* sp., *Pheidole* sp., *Wasmannia auropunctata, Solenopsis* sp. | Ponerinae Formicinae, Myrmicinae, | Phytophagous on plants / trophobiotic, facultative, including *N. villosa* | Costa Rica | 6, 7 |
| Eurybiini: Mesosemiina | | | | | |
| *Hyphilaria thasus* (Stoll, 1780) | *Camponotus* spp. | Formicinae | Phytophagous on plants / non-trophobiotic, caterpillars may be living in places with ants, but do not establish stable associations | Costa Rica, Trinidad, Colombia, Brazil | 1, 9, 10 |
| Nymphidiini: Zabuellina | | | | | |
| *Zabuella paucipuncta* (Spitz, 1930) | *Crematogaster* sp. | Myrmicinae | Phytophagous on plant / trophobiotic, obligate | Brazil | 11, 12 |
| Nymphidiini: Stalachtina | | | | | |
| *Protonymphidia senta* (Hewitson, 1853) | *Pheidole* *biconstricta* complex, *Ph. gertrudae* | Myrmicinae | Phytophagous on plant / trophobiotic, specialized on *Pheidole­-Bauhinia* association | Ecuador, Brazil | 1, 6, 13, 14 |
| *Stalachtis phlegia* (Cramer, 1779) | *Camponotus* spp., *Crematogaster* sp., *Cephalotes* sp., *Ectatomma* sp. | Formicinae, Myrmicinae, Ectatomminae | Phytophagous on plant / trophobiotic, facultative | Brazil | 1, 15 |
| Nymphidiini: Pachythonina | | | | | |
| **Pachythone gigas* Godman & Salvin, 1878 | *Azteca* sp. | Dolichoderinae | Exudates and predation on HPHs / non-trophobiotic, obligate | Costa Rica | 16 |
| **Pachythone xanthe* Bates 1868 | *Azteca* cf. *chartifex* | Dolichoderinae | Predation on HPHs / non-trophobiotic, obligate | Brazil | 17 |
| **Pseudonymphidia agave (Godman & Salvin, 1886) | *Neoponera villosa* | Ponerinae | Unknown / possibly social parasite, caterpillars with specialized morphology found inside the ant-nest with the brood | Mexico | This study |
| *Minstrellus grandis* (Callaghan, 1999) | *Pseudomyrmex triplarinus* group | Pseudomyrmecinae | Unknown / ant-mediated oviposition, immatures unknown | Brazil | 18 |
| Nymphidiini: Theopina | | | | | |
| Theope archimedes zyzyxoxyx D’Abrera, 1994 | *Dolichoderus bispinosus* | Dolichoderinae | Phytophagous on plant / trophobiotic, obligate | Panama | 6 |
| *Theope bacenis* Schaus, 1890 | *Azteca* sp. | Dolichoderinae | Phytophagous on plant / trophobiotic, obligate | Costa Rica | 6, 7 |
| Theope eudocia Westwood, 1851 | *Azteca* sp. | Dolichoderinae | Phytophagous on plant / trophobiotic, obligate | Brazil | 14, 19, 20, 21 |
| *Theope guillaumei cecropia* DeVries & Hall, 1996 | *Solenopsis* (*diplorhoptrum* group) | Myrmicinae | Phytophagous on plant / trophobiotic, probably facultative | Costa Rica | 6, 7, 22, 23 |
| Theope leucanthe Bates, 1868 | *Azteca* sp. | Dolichoderinae | Phytophagous on plant / trophobiotic, obligate | Brazil | 14 |
| *Theope lycaenina* Bates, 1868 | *Azteca* *chartifex*, *Azteca* spp. | Dolichoderinae | Phytophagous, pupation inside ant-built shelters on plants / trophobiotic, obligate | Panama, Costa Rica, Brazil | 6, 7, 14, 24 |
| Theope phaeo Prittwitz, 1865 | *Azteca* sp. | Dolichoderinae | Phytophagous on plant / trophobiotic, obligate | Costa Rica | 19 |
| Theope pieridoides Felder & Felder, 1865 | *Azteca* sp. | Dolichoderinae | Phytophagous on plant / trophobiotic, obligate | Brazil | 14 |
| *Theope publius* *publius* Felder & Felder, 1861 | *Azteca* sp. | Dolichoderinae | Phytophagous on plant / trophobiotic, obligate | Costa Rica | 7, 19 |
| Theope terambus (Godart, 1824) | *Azteca* sp., *Pheidole* sp. | Dolichoderinae, Myrmicinae | Phytophagous, pupation inside ant-built shelters on plants / trophobiotic, obligate | Brazil | 14, 25 |
| *Theope thestias* Hewitson, 1860 | *Camponotus blandus, Ca. crassus, Ca. melanoticus, Ca. mus, Ca. rufipes, Ca. sericeiventris, Cephalotes pusillus* | Formicinae, Myrmicinae | Phytophagous on plant / trophobiotic, facultative | Brazil | 14 |
| *Theope virgilius* (Fabricius, 1793) | *Azteca* sp. | Dolichoderinae | Phytophagous on plant / trophobiotic, obligate | Costa Rica, Panama | 6, 7 |
| Nymphidiini: Nymphidiina | | | | | |
| *Catocyclotis adelina* (Butler, 1872) | *Myrmelachista* sp. | Formicinae | Phytophagous on plant / trophobiotic, degree unknown | Costa Rica | 26 |
| *Catocyclotis densemaculata* (Hewitson, 1870) | *Crematogaster* sp. | Myrmicinae | Phytophagous on plant / trophobiotic, degree unknown | Peru | 27 |
| *Nymphidium a. azanoides* Butler, 1867 | *Pheidole* sp., *Ph. biconstricta*, *Wasmannia* sp. | Myrmicinae | Phytophagous on plant / trophobiotic, facultative | Costa Rica, Panama, Brazil | 1, 6, 7 |
| *Nymphidium cachrus* (Fabricius, 1787*)* | *Paratrechina* sp., *Crematogaster* sp., *Pheidole* sp., *Megalomyrmex foreli, Solenopsis* sp. | Formicinae, Myrmicinae | Phytophagous on plant / trophobiotic, facultative | Costa Rica, Panama, Ecuador | 6, 7, 28 |
| Nymphidium caricae (Linnaeus, 1758) | *Crematogaster* sp., *Pheidole* sp*., Ph.* nr. *biconstricta*, *Megalomyrmex foreli*, *Ectatomma tuberculatum* | Myrmicinae | Phytophagous on plant / trophobiotic, facultative | Ecuador, Brazil, French Guiana | 1, 6, 28, 29 |
| *Nymphidium haematostictum* Godman & Salvin, 1878 | *Crematogaster* sp. | Myrmicinae | Phytophagous on plant / trophobiotic, degree unknown | Costa Rica, Panama | 6, 7 |
| Nymphidium leucosia (Hübner, 1806) | *Brachymyrmex* sp., *Camponotus* sp., *Crematogaster* sp., *Ochetomyrmex* sp., *Gnamptogenys* sp. | Formicinae, Myrmicinae, Ectatomminae | Phytophagous on plant / trophobiotic, facultative | Ecuador, Brazil | 1, 6 |
| Nymphidium lisimon (Stoll, 1790) | *Wasmannia auropunctata* | Myrmicinae | Phytophagous on plant / trophobiotic, obligate | Colombia, Ecuador, Brazil | 1, 30 |
| *Nymphidium mantus* (Cramer, 1775) | *Azteca* sp. | Dolichoderinae | Phytophagous on plant / trophobiotic, probably obligate | Costa Rica, Panama, Guyana | 6, 7, 28 |
| Nymphidium minuta H. Druce, 1904 | *Crematogaster* sp. | Myrmicinae | Phytophagous on plant, facultative carnivory on ant-tended treehoppers / trophobiotic, probably obligate | Ecuador, Guyana, Brazil | 28 |
| Nymphidium onaeum Hewitson, 1869 | *Pheidole* sp. *Solenopsis* sp. | Myrmicinae | Phytophagous on plant / trophobiotic, facultative | Costa Rica, Panama | 6, 7, 19 |
| Nymphidium velatum Stichel, 1914 (referred to as N. nr derufata) | *Megalomyrmex foreli* | Myrmicinae | Phytophagous on plant / trophobiotic, degree unknown | Ecuador | 6 |
| Nymphidiini: Pandemina | | | | | |
| *Parvospila cilissa* (Hewitson, 1863) | *Tapinoma* sp., *Wasmannia auropunctata, Crematogaster* sp., *Cr. brevispina*, *Ectatomma ruidum* | Dolichoderinae, Myrmicinae, Ectatomminae | Phytophagous on plant / trophobiotic, facultative | Costa Rica | 6, 7, 28 |
| *Parvospila emylius* (Cramer, 1775) | *Crematogaster* sp*.,* *Solenopsis* sp., *Ectatomma tuberculatum* | Myrmicinae, Ectatomminae | Phytophagous on plant / trophobiotic, facultative | Ecuador, Guyana, Brazil | 1, 6, 28 |
| **Setabis lagus jansoni* (Butler, 1870) | *Pheidole* sp., *Ph. biconstricta, Azteca* sp. | Dolichoderinae, Myrmicinae | Predation on HPHs, caterpillar and pupation inside ant-built shelters on plants / non-trophobiotic, obligate | Costa Rica | 6, 7, 28 |
| **Setabis lagus lagus* (Cramer, 1777) | *Pheidole biconstricta* | Myrmicinae | Predation on HPHs, myrmecophagy in laboratory conditions, and extrafloral secretions, caterpillars inside ant-built shelters on plants / non-trophobiotic, obligate | Brazil | 28, 31 |
| Nymphidiini: Lemoniadina | | | | | |
| Annulata annulifera (Godman, 1903) | *Pheidole* sp., *Megalomyrmex balzani*, *Paraponera clavata*, *Ectatomma tuberculatum* | Myrmicinae, Paraponerinae, Ectatomminae | Bamboo extrafloral secretions, adults observed stealing nectar from the mandibles of *E. tuberculatum* / trophobiotic, facultative | Peru | 32 |
| *Periplacis felsina* (Hewitson, 1863) | *Camponotus crassus* | Formicinae | Phytophagous on plant / trophobiotic, facultative | Brazil | 33 |
| *Periplacis menander* (Stoll, 1780) | *Camponotus* sp., *Crematogaster* sp. | Formicinae, Myrmicinae | Phytophagous on plant / trophobiotic, facultative | Costa Rica, Panama, Ecuador | 6, 7 |
| *Periplacis pretus* (Cramer, 1777) | *Azteca* sp., *Camponotus* sp., *Crematogaster* sp. | Dolichoderinae, Formicinae, Myrmicinae | Phytophagous on plant / trophobiotic, facultative | Costa Rica | 6, 7, 34 |
| *Synargis abaris* (Cramer, 1776) | *Camponotus femoratus, Pheidole* nr*. biconstricta, Ectatomma tuberculatum* | Formicinae, Myrmicinae, Ectatomminae | Phytophagous on plant / trophobiotic, facultative | Brazil, Ecuador | 1, 6 |
| Synargis calyce (Felder & Felder, 1862) | *Azteca* sp*., Camponotus blandus, Ca. crassus, Ca. rufipes, Ca. sericeiventris, Ca. melanoticus, Cephalotes pusillus, Ectatomma tuberculatum* | Dolichoderinae, Formicinae, Myrmicinae, Ectatomminae | Phytophagous on plant / trophobiotic, facultative | Brazil | 1, 35, 36 |
| *Synargis galena* (Bates, 1868  *Synargis gela* (Hewitson, 1853) | *E. brunneum, E. tuberculatum*  *Ectatomma tuberculatum* | Ectatomminae  Ectatomminae | Phytophagous on plant / trophobiotic, facultative  Phytophagous on plant / trophobiotic, obligate | Brazil  Ecuador, Brazil | 1, 37  1, 6 |
| *Synargis mycone* (Hewitson, 1865) | *Ectatomma ruidum*, *E. tuberculatum*, *Camponotus sericeiventris*, *Cephalotes atratus, Wasmannia* sp. | Ectatomminae, Formicinae, Myrmicinae | Phytophagous on plant / trophobiotic, facultative | Costa Rica | 6, 7 |
| *Synargis palaeste* (Hewitson, 1870) | *Camponotus sericeiventris* | Formicinae | Unknown / Unknown, prepupae found at the nest entrance | Costa Rica | 7 |
| *Juditha caucana* (Stichel, 1911) | *Dolichoderus bispinosus*, *Camponotus* sp. (need confirmation) | Dolichoderinae, Formicinae | Phytophagous on plant / trophobiotic, obligate | Belize, Costa Rica, Panama, Colombia | 6, 7, 19, 22, 38, 39 |
| *Juditha dorilis* (Bates, 1866) | *Dolichoderus validus* | Dolichoderinae | Unknown / Ant-hemipteran oviposition | Costa Rica | 6, 7 |
| *Juditha molpe* (Hübner, 1808) | *Dolichoderus* *bispinosus* | Dolichoderinae | Phytophagous on plant / trophobiotic, obligate | Trinidad, Brazil | 1, 6, 20 |
| *Juditha odites praeclarum* (Bates 1866) | *Dolichoderus bispinosus* | Dolichoderinae | Aphytophagy indicated / Ant-hemipteran oviposition | Panama | 6, 7 |
| *Thisbe irenea* (Stoll, 1780) | *Dolichoderus* sp., *Camponotus* sp., *Ca. sericeiventris*, *Paratrechina* sp., *Aphaenogaster* *araneoides*, *Pheidole* sp.*, Solenopsis* (*diplorhoptrum* group), *S. geminata*, *Ectatomma ruidum*, *E. tuberculatum* | Dolichoderinae, Formicinae, Myrmicinae, Ectatomminae | Phytophagous on plant / trophobiotic, facultative | Belize, Costa Rica, Panama, Ecuador, Brazil | 1, 6, 7 |
| *Thisbe silvestre* Kaminski, Iserhard & Freitas 2015 | *Camponotus* sp., *Ca. rufipes*, *Procryptocerus* sp., *Pheidole* sp. | Formicinae, Myrmicinae | Phytophagous on plant / trophobiotic, facultative | Brazil | 40 |
| *Lemonias caliginea* (Butler, 1867) | *Camponotus atriceps* | Formicinae | Phytophagous on plant / trophobiotic, facultative, pupation in ant-built shelters at the base of host plant | Mexico | 41 |
| *Lemonias egaensis* (Butler, 1867) | *Camponotus distinguendus*, *Camponotus* sp. | Formicinae | Phytophagous on plant / trophobiotic, facultative | Ecuador, Brazil | 1, 6 |
| Lemonias zygia (Hübner, 1807) | *Camponotus blandus, Ca. crassus, Ca. melanoticus, Ca. rufipes* | Formicinae | Phytophagous on plant / trophobiotic, facultative | Brazil | 1, 42 |
| ***Aricoris arenarum* (Schneider, 1937) | *Camponotus punctulatus* | Formicinae | Hemiptera honeydew and ant trophallaxis / Social parasite within ant shelters | Argentina,  Brazil, Uruguay | 6, 43, 44 |
| *Aricoris chilensis* (C. Felder & R. Felder, 1865) | *Camponotus punctulatus*, *Ca. mus* | Formicinae | Phytophagous on plant / trophobiotic, ant-mediated oviposition, rest in underground shelter built by ants | Argentina | 1, 43, 45, 46, 47 |
| *Aricoris domina* (Bates, 1865) | *Ectatomma* *tuberculatum*, *Ectatomma* sp. | Ectatomminae | Unknown / oviposition near ant-tended hemipterans | Panama | 48 |
| *Aricoris erostratus* (Westwood, 1851*)* | *Camponotus* spp., *Paratrechina longicornis* | Formicinae | Phytophagous on plant / trophobiotic, ant-mediated oviposition, rest in underground shelter built by ants | Colombia | 1, 49 |
| *Aricoris notialis* (Stichel, 1910) | *Camponotus punctulatus* | Formicinae | Phytophagous on plant / trophobiotic, ant-mediated oviposition, rest in underground shelter built by ants | Argentina, Brazil, Uruguay | 1, 43 |
| *Aricoris propitia* (Stichel, 1910) | *Solenopsis saevissima* complex | Myrmicinae | Phytophagous on plant / trophobiotic, ant-mediated oviposition, rest in underground shelter built by ants | Brazil | 50 |
| *Aricoris signata* (Stichel, 1910) | *Solenopsis saevissima richteri* | Myrmicinae | Phytophagous on plant / trophobiotic, ant-mediated oviposition, rest in underground shelter built by ants | Argentina | 51 |
| **Unidentified Nymphidiini | *Azteca* sp. | Dolichoderinae | Unknown / Photo taken in the ant nest | Costa Rica | 52 |

**References**

1. L. A. Kaminski, unpublished data.
2. DeVries, P. J. & Penz, C. M. Entomophagy, behavior, and elongated thoracic legs in the myrmecophilous Neotropical butterfly *Alesa amesis* (Riodinidae). *Biotropica* **32**, 712–721 (2000).
3. DeVries, P. J. & Penz, C. M. Early stages of the entomophagous metalmark butterfly *Alesa amesis* (Riodinidae: Eurybiini). *J. Lepid. Soc.* **56**, 265–2712 (2002).
4. Gallard J-Y. 2017. Les Riodinidae de Guyane. *Pensoft, Sofia* (2017).
5. Horvitz, C. C., Turnbull, C. & Harvey, D. J. Biology of immature *Eurybia elvina* (Lepidoptera: Riodinidae), a myrmecophilous metalmark butterfly. *Ann. Entomol. Soc. Am.* **80**, 513–519 (1987).
6. DeVries, P. J., Chacon, I. A. & Murray, D. Toward a better understanding of host use and biodiversity in riodinid butterflies (Lepidoptera). *J. Res. Lepid.* **31**, 103–126 (1992).
7. DeVries PJ. 1997. The butterflies of Costa Rica and their natural history. Vol II: Riodinidae. *Princeton University Press, Princeton* (1997).
8. Travassos, M. A., DeVries, P. J. & Pierce, N. E. A novel organ and mechanism for larval sound production in butterfly caterpillars: *Eurybia elvina* (Lepidoptera: Riodinidae). *Trop. Lepid.* **18**, 20–23 (2008).
9. Barcant, M. Butterflies of Trinidad and Tobago. *Collins, London* (1970).
10. Brévignon, C. Élevage de deux Riodininae guyanais, *Napaea beltiana* Bates et *Cremna thasus* Stoll. 1. À propos de la myrmécophilie des chenilles (Lep. Lycaenidae Riodininae). *Alexanor* **17**, 403–413 (1992).
11. DeVries, P. J., Cabral, B. C. & Penz, C. M. The early stages of *Apodemia paucipuncta* (Riodinidae): myrmecophily, a new caterpillar ant-organ and consequences for classification. *Milw. Public Mus. Contrib. Biol. Geol.* **102**, 1–13 (2004).
12. Kaminski, L. A. Polyphagy and obligate myrmecophily in the butterfly *Hallonympha paucipuncta* (Lepidoptera: Riodinidae) in the Neotropical Cerrado Savanna. *Biotropica* **40**, 390–394 (2008).
13. Hall, J. P. W. Two new genera in the Neotropical riodinid tribe Nymphidiini (Riodinidae). *J. Lepid. Soc.* **54**, 41–46 (2000).
14. Kaminski, L. A., Mota, L. L., Freitas, A. V. L. & Moreira, G. R. P. Two ways to be a myrmecophilous butterfly: natural history and comparative immature-stage morphology of two species of *Theope* (Lepidoptera: Riodinidae). *Biol. J. Linn. Soc.* **108**, 844–870 (2013).
15. Brown, K. S. Jr. Neotropical Lycaenidae: an overview. In: New, T. R. (ed.) Conservation biology of Lycaenidae (Butterflies). *IUCN, Gland*, pp 45–61 (1993).
16. Moraga Medina, R. 2014. *Pachythone gigas* (Riodinidae). Área de Conservación Guanacaste (2014). <https://www.acguanacaste.ac.cr/paginas-de-especies/insectos/111-160riodinidae/581-i-pachythone-gigas-i-riodinidae>
17. Mota, L. L., Kaminski, L. A. & Freitas, A. V. L. The tortoise caterpillar: carnivory and armoured larval morphology of the metalmark butterfly *Pachythone xanthe* (Lepidoptera: Riodinidae). *J. Nat. Hist.* **54**, 309–319 (2020).
18. Kaminski, L. A., Carneiro, E., Dolibaina, D. R., Casagrande, M. M. & Mielke, O. H. H. Oviposition of *Minstrellus grandis* (Lepidoptera: Riodinidae) in a harmful ant-plant symbiosis. *Acta Amazon.* **50**, 256–259 (2020).
19. Janzen, D. H. & Hallwachs, W. Dynamic database for an inventory of the macrocaterpillar fauna, and its food plants and parasitoids, of Area de Conservación Guanacaste (ACG), northwestern Costa Rica (2020). Available from: http://janzen.sas.upenn.edu (accessed 16 December 2020).
20. Guppy J. Notes on the habits and early stages of some Trinidad butterflies. Trans. Entomol. Soc. London **1904**, 225–228 (1904).
21. Kirkpatrick, T. W. Notes on minor insect pests of cacao in Trinidad. Part 2. Lepidoptera (butterflies and moths). In: A report on cacao research 1953. *Imperial College of Tropical Agriculture, St. Augustine, Trinidad and Tobago*, pp 67–72 (1954).
22. Longino, J. T. *Azteca* ants in *Cecropia* trees: taxonomy, colony structure, and behaviour. In: Huxley, C. R. & Cutler, D. F. (eds.) Ant–Plant Interactions. Oxford University Press, Oxford, pp 271–288 (1991).
23. DeVries, P. J. & Hall, J. P. W. Two new species of Costa Rican butterflies (Lepidoptera: Riodinidae). *Trop. Lepid.* **7**, 87–90 (1996).
24. Freitas, A. V. L. Local polyphagy in *Theope lycaenina* Bates, 1868 (Riodinidae: Nymphidiini). *J. Lepid. Soc.* **65**, 264–265 (2011).
25. Nobre, C. E. B. & Schlindwein, C. New records for species of *Theope* (Lepidoptera, Riodinidae) for the state of Pernambuco and northeastern Brazil, with notes on their natural history. *Rev. Bras. Entomol.* **55**, 275–278 (2011).
26. K. Nishida in: Penz, C. M. & DeVries, P. *Catocyclotis aemulius adelina* (Riodinidae) revisited: it ain’t necessarily so. *J. Lepid. Soc.* **58**, 178–182 (2004), and pers. comm.
27. Aibar-Abregú, P. Hostplant records for the myrmecophilous butterfly *Harveyope densemaculata* (Hewitson, 1870) (Lepidoptera: Riodinidae). *Trop. Lepid. Res.* **24**, 121 (2014).
28. Hall, J. P. W. A monograph of the Nymphidiina (Lepidoptera: Riodinidae: Nymphidiini): Phylogeny, taxonomy, biology, and biogeography. *The Entomological Society of Washington, Washington* (2018).
29. Brévignon, C. & Gallard, J.-Y. Inventaire des Riodinidae de Guyane Française. VI - Riodininae: Nymphidiini, Stalachtini. Description de nouveaux taxa – Première partie - (Lepidoptera). *Lambillionea* **99**, 91–100 (1999).
30. Callaghan, C. J. Notes on the biology of three Riodinine species: *Nymphidium lisimon attenuatum*, *Phaenochitonia sagaris satnius*, and *Metacharis ptolomaeus* (Lycaenidae: Riodininae). *J. Res. Lepid.* **27**, 109–114 (1989).
31. Kaminski, L. A. & Dias Lima, L. Larval omnivory in the myrmecophilous butterfly *Setabis lagus lagus* (Riodinidae: Nymphidiini). *J. Lepid. Soc.* **73**, 276–279 (2019).
32. Torres, P. J. & Pomerantz, A. F. Butterfly kleptoparasitism and first account of immature stages, myrmecophily, and bamboo host plant of the metalmark *Adelotypa annulifera* (Riodinidae). *J. Lepid. Soc.* **70**, 130–138 (2016).
33. Callaghan, C. J. Studies on Restinga butterflies: I. Life cycle and immature biology of *Menander felsina* (Riodinidae), a myrmecophilous metalmark. *J. Lepid. Soc.* **31**, 173–182 (1977).
34. Donahue, J. P. Strategies for survival. The cause of a caterpillar. *Terra* **17**, 3–9 (1979).
35. Callaghan, C. J. Restinga butterflies: Biology of *Synargis brennus* (Stichel) (Riodinidae). *J Lepid. Soc.* **40**, 93–96 (1986).
36. Alves-Silva, E., Bächtold, A. & Del-Claro K. Florivorous myrmecophilous caterpillars exploit an ant–plant mutualism and distract ants from extrafloral nectaries. *Austral Ecol.* **43**, 643–650 (2018).
37. Kaminski, L. Formigas, besouros e lepidópteros. In: Diehl, E. (ed.) Interações das formigas com outros organismos: diversidade ecológica e evolutiva. *Oikos Ltda., São Leopoldo, Brasil*, pp 51–65 (2017).
38. Callaghan, C. J. Notes on the immature biology of two myrmecophilous Lycaenidae: *Juditha molpe* (Riodininae) and *Panthiades bitias* (Lycaeninae). *J. Res. Lepid.* **20**, 36–42 (1982).
39. Hall, J. P. W. & Harvey, D. J. A phylogenetic analysis of the Neotropical riodinid butterfly genera *Juditha*, *Lemonias*, *Thisbe* and *Uraneis*, with a revision of *Juditha* (Lepidoptera: Riodinidae: Nymphidiini). *Syst. Entomol.* **26**, 453–490 (2001).
40. Kaminski, L. A., Iserhard, C. A. & Freitas, A. V. L. *Thisbe silvestre* sp. nov. (Lepidoptera: Riodinidae): a new myrmecophilous butterfly from the Brazilian Atlantic Forest. *Austral Entomol.* **55**, 138–146 (2016).
41. Ross, G. N. Life history studies on Mexican butterflies. II. Early stages of *Anatole rossi* a new myrmecophilous metalmark. *J. Res. Lepid.* **3**, 81–94 (1964).
42. Zikán, J. F. Beiträge zur Biologie von 19 Riodiniden-Arten (Riodinidae - Lepidoptera). *Dusenia* **4**, 403–413 (1953).
43. Volkmann, L. & Núñez-Bustos, E. Mariposas Serranas de Argentina Central. Tomo 1. Papilionidae, Pieridae, Lycaenidae, Riodinidae. *Equipo Grafico, Huerta Grande, Cordoba, Argentina* (2010).
44. Kaminski, L. A., Volkmann, L., Callaghan, C. J., DeVries, P. J. & Vila, R. The first known riodinid ‘cuckoo’ butterfly reveals deep-time convergence and parallelism in ant social parasites. Zool. J. Linn. Soc. (2021) <https://doi.org/10.1093/zoolinnean/zlaa150>.
45. Bourquin, F. Notas sobre la metamorfosis de *Hamearis susanae* Orfila, 1953, con oruga mirmicófila (Lep. Riodin.). *Rev. Soc. Entomol. Argent.* **16**, 83–87 (1953).
46. Hayward, K. J. Datos para el estudio de la ontogenia de lepidópteros argentinos. *Miscelánea. Inst. Miguel Lillo, Univ. Nac. Tucumán* **31**, 1–142 (1969).
47. Canals, G. R. Mariposas de Misiones. *L.O.L.A., Buenos Aires* (2003).
48. Robbins, R. K. & Aiello, A. Foodplant and oviposition records for Panamanian Lycaenidae and Riodinidae. *J. Lepid. Soc.* **36**, 65–75 (1982).
49. Schremmer, F. Zur Bionomie und Morphologie der myrmekophilen Raupe und Puppe der neotropischen tagfalter-art *Hamearis erostratus* (Lepidoptera: Riodinidae). *Entomol. Ger.* **4**, 113–121 (1978).
50. Kaminski, L. A. & Carvalho-Filho, F. S. Life history of *Aricoris propitia* (Lepidoptera: Riodinidae)—A myrmecophilous butterfly obligately associated with fire ants. *Psyche* **2012**, Article ID 126876 (2012).
51. Bruch, C. Orugas mirmecofilas de *Hameris epulus signatus* - Stich. *Rev. Soc. Entomol. Argent.* **1**, 2–9 (1926).
52. J. Longino in: Hall, J. P. W., Harvey, D. J. & Janzen, D. H. Life history of *Calydna sturnula* with a review of larval and pupal balloon setae in the Riodinidae (Lepidoptera). *Ann. Entomol. Soc. Am.* **97**, 310–321 (2004).

**Table S2**. Sample information of relevant taxa in Fig. 2.

| **Sample ID** | **Species** | **Stage** | **Sample location** | **Previously published by** | **Barcode accession number** |
| --- | --- | --- | --- | --- | --- |
| JH03R005 | *Pixus corculum* | Adult | El Durango, Ecuador | 1 | [KT286472](http://www.ncbi.nlm.nih.gov/entrez/query.fcgi?cmd=Search&db=nucleotide&term=KT286472%5Baccn%5D&doptcmdl=GenBank) |
| BLU826 | *Pachythone xanthe* | Adult | Alta Floresta - MT, Brazil | 2 |  |
| KP849182 | *Pachythone gigas* | Adult | Barro Colorado Island, Panama | 3 | KP849182 |
| NS0362 | *Roeberella lencates* | Adult | Recife - PE, Brazil | 4 | [MG607921](http://www.ncbi.nlm.nih.gov/entrez/query.fcgi?cmd=Search&db=nucleotide&term=MG607921%5Baccn%5D&doptcmdl=GenBank) |
| LLM517 | *Archaeonympha* sp. | Adult | Alta Floresta - MT, Brazil | unpublished |  |
| JH03R008 | *Pseudonymphidia agave* | Adult | El Durango, Ecuador | 1 | [KT286306](http://www.ncbi.nlm.nih.gov/entrez/query.fcgi?cmd=Search&db=nucleotide&term=KT286306%5Baccn%5D&doptcmdl=GenBank) |
| MAL-05053 | *Pseudonymphidia agave* | Adult | Calakmul, Campeche, Mexico | 5 |  |
| MAL-05054 | *Pseudonymphidia agave* | Adult | Calakmul, Campeche, Mexico | 5 |  |
| CH0000657-A01 | *Pseudonymphidia agave* | Larva | Ejido Blasillo, Campeche, Mexico | target sequence | MW627452 |

**References**

1. Espeland, M. *et al*. Ancient Neotropical origin and recent recolonisation: Phylogeny, biogeography and diversification of the Riodinidae (Lepidoptera: Papilionoidea). *Mol. Phylogenet. Evol.* **93**, 296–306 (2015).
2. N. Seraphim, unpubl. data.
3. Basset, Y. *et al*. The butterflies of Barro Colorado Island, Panama: Local extinction since the 1930s. *PLoS ONE* **10**, e0136623 (2015).
4. Seraphim, N. *et al*. Molecular phylogeny and higher systematics of the metalmark butterflies (Lepidoptera: Riodinidae). *Syst. Entomol.* **43**, 407–425 (2018).
5. C. Pozo *et al.*, unpubl. data.

**Table S3.** Number of specimens in data bases of collections housing *Pseudonymphidia agave* specimens from Mexico.

| **Entomological Collection source** | **Nb. of specimens** |
| --- | --- |
| AMNH Entomology Collection (New York, NY, USA) | 8 |
| CMNH Arthropods Collection (Cleveland, OH, USA) | 1 |
| CNIN Colección Nacional de Insectos (Instituto de Biología, UNAM) (Mexico, DF, Mexico) | 15 |
| ECO-CH-L Colección Lepidopterológica (Chetumal, QRoo, Mexico) | 3 |
| FSMC Collection of Entomology (Gainesville, FL, USA) | 2 |
| MGCL-FLMNH Entomological Collection (Gainesville, FL, USA) | 13 |
| MZFC Colección Lepidopterológica (Facultad de Ciencias UNAM) (Mexico, DF, Mexico) | 1 |
| USNM National Entomological Collection (Washington, DC, USA) | 5 |
| Reports without collecting information | 4 |
| **Total** | **52** |

**Table S4.** Number of specimens and Mexican states where *Pseudonymphidia agave* individuals have been collected.

| **State** | **Nb. of specimens** |
| --- | --- |
| Campeche | 2 |
| Chiapas | 3 |
| Colima | 1 |
| Oaxaca | 4 |
| Puebla | 2 |
| Quintana Roo | 2 |
| San Luis Potosí | 1 |
| Veracruz | 37 |
| **Total** | **52** |

**Fig. S1.** Flight activity according to records of Mexican specimens in Museums.
